# Pharmacological effects and mechanisms of medicine food homology species and active ingredients in ameliorating ovarian aging

**DOI:** 10.3389/fnut.2025.1756703

**Published:** 2026-01-21

**Authors:** Jiayi Chen, Xiaotian Li, Xinle Lai, Ruoyi Xu, Zheqi Liu, Jia Xing, Liuqing Yang, Qin Zhang

**Affiliations:** 1 Department of TCM Gynecology, Hangzhou TCM Hospital Affiliated to Zhejiang Chinese Medical University, Hangzhou, China; 2 Research Institute of Women's Reproductive Health, Zhejiang Chinese Medical University, Hangzhou, China; 3 Zhejiang Key Laboratory of Precise Protection and Promotion of Fertility, Hangzhou, China

**Keywords:** active ingredients, herbal extracts, medicine food homology, ovarian aging, traditional Chinese medicine

## Abstract

Ovarian aging is the process of decline in ovarian reserve, endocrine function with age, leading to reduced fertility and increased risk of various related diseases. In recent years, medicine food homology (MFH) species have attracted much attention for their potential to delay ovarian aging due to their dietary and medicinal values. In this review, we have focused on the intervention of MFH species and active ingredients on ovarian aging, with an emphasis on the molecular mechanisms involved in antioxidant, anti-inflammatory, apoptosis inhibitory, balance of autophagy, maintenance of genome stability, mitochondrial function protective and estrogen-like effects through multiple signaling pathways (e.g., PI3K/Akt, Nrf2/HO-1, SIRT1/mTOR, Nrf2/ARE, etc.). Possessing the characteristics of multi-pathway and multi-target effects, MFH species and active ingredients provide new ideas for the research and development of health food and functional preparations. High-quality clinical studies are still needed for verification.

## 1 Introduction

Ovarian aging refers to the gradual deterioration of ovarian function, primarily characterized by a decline in the quantity and quality of oocytes and a reduction in reproductive function (1). This process can be categorized into physiological and pathological aging: the former refers to the natural decline in ovarian function with age until menopause (2); the latter includes premature ovarian failure (POF), premature ovarian insufficiency (POI), decline in ovarian reserve function (DOR), which are often triggered by genetic factors, enzyme deficiencies, immune abnormalities, environmental exposure, and surgical trauma (3–5). In recent years, with the rapid socio-economic development and changes in women’s lifestyles, the phenomenon of delayed marriage and childbearing has become increasingly common. Consequently, ovarian aging has shown a tendency to occur earlier and progress more rapidly, resulting in a significant decline in fertility, especially after the age of 35 (6). Data indicate that the global prevalence of POI among women has reached 3.5% (7), and is closely associated with infertility, preeclampsia, embryonic chromosomal abnormalities, and a range of complications, including cardiovascular disease, osteoporosis, depression, and cognitive dysfunction (1, 8–11).

Ovarian aging involves multiple physiological, molecular and cellular processes, including apoptosis and autophagy, oxidative stress, mitochondrial dysfunction, chronic inflammation and genome instability (12). However, societal awareness and prevention of ovarian aging remain inadequate (13), and effective intervention strategies are urgently needed to maintain or restore oocyte quality and ovarian function, thereby extending the female reproductive window and improving overall quality of life.

The concept of Medicine Food Homology (MFH) is derived from the *Huang Di Nei Jing*: ‘consumption on an empty stomach serves as food, while administration to the patient functions as medicine.’ This principle reflects the traditional view that food and medicine share a common origin and can be used interchangeably to restore physiological balance and regulate metabolic disorders. In a modern biomedical context, MFH broadly refers to edible botanical resources with dual nutritional and therapeutic properties, conceptually overlapping with internationally recognized categories such as functional foods, nutraceuticals, and medicinal dietary plants. As public health and nutritional awareness increase, national regulations have been established to systematically identify traditional Chinese medicinal herbs with MFH properties and incorporate them into the List of Items that are Both Food and Medicine, clarifying their scope of application and safety assessment criteria. As of 2024, more than 100 traditional Chinese medicinal substances have been formally included in the official Catalogue of Substances Traditionally Used as Both Food and Chinese Medicinal Materials and its subsequent updates, such as *Panax ginseng* C. A. Mey., *Lycium barbarum* L., Angelica sinensis (Oliv.) Diels; these herbs are rich in saponins, flavonoids, polysaccharides, alkaloids, polyphenols, and other active ingredients (14–17). They are characterized by multi-pathway and multi-target effects, low adverse reactions, and suitability for long-term consumption (18), demonstrating superior functionality and safety compared to traditional single-chemistry medicines or ordinary food. In recent years, MFH species have achieved positive results in the fields of diabetes (19), oncology (20), Alzheimer’s disease (21) and immunomodulation (22), reflecting their growing research interest in age-related conditions across different physiological systems. Notably, accumulating evidence indicates that ovarian aging shares common molecular features with metabolic and chronic degenerative diseases, particularly in redox imbalance, mitochondrial dysfunction, and disrupted energy metabolism (23). Therefore, MFH species have gradually become a focus in ovarian aging research, as they may exert beneficial regulatory effects through antioxidant, anti-inflammatory, and estrogen-like effects, as well as endocrine and immune modulation and the promotion of cellular repair.

Based on the aforementioned background, this review aims to systematically summarize the research progress of MFH species and their active ingredients in delaying or improving ovarian aging, to focus on their main pharmacological effects and molecular mechanisms, and to explore the potential and challenges of their future clinical applications and the development of functional foods, thereby providing additional insights and references for the prevention and treatment of ovarian aging.

## 2 Mechanisms of ovarian aging

### 2.1 Oxidative stress

**Figure 1:**
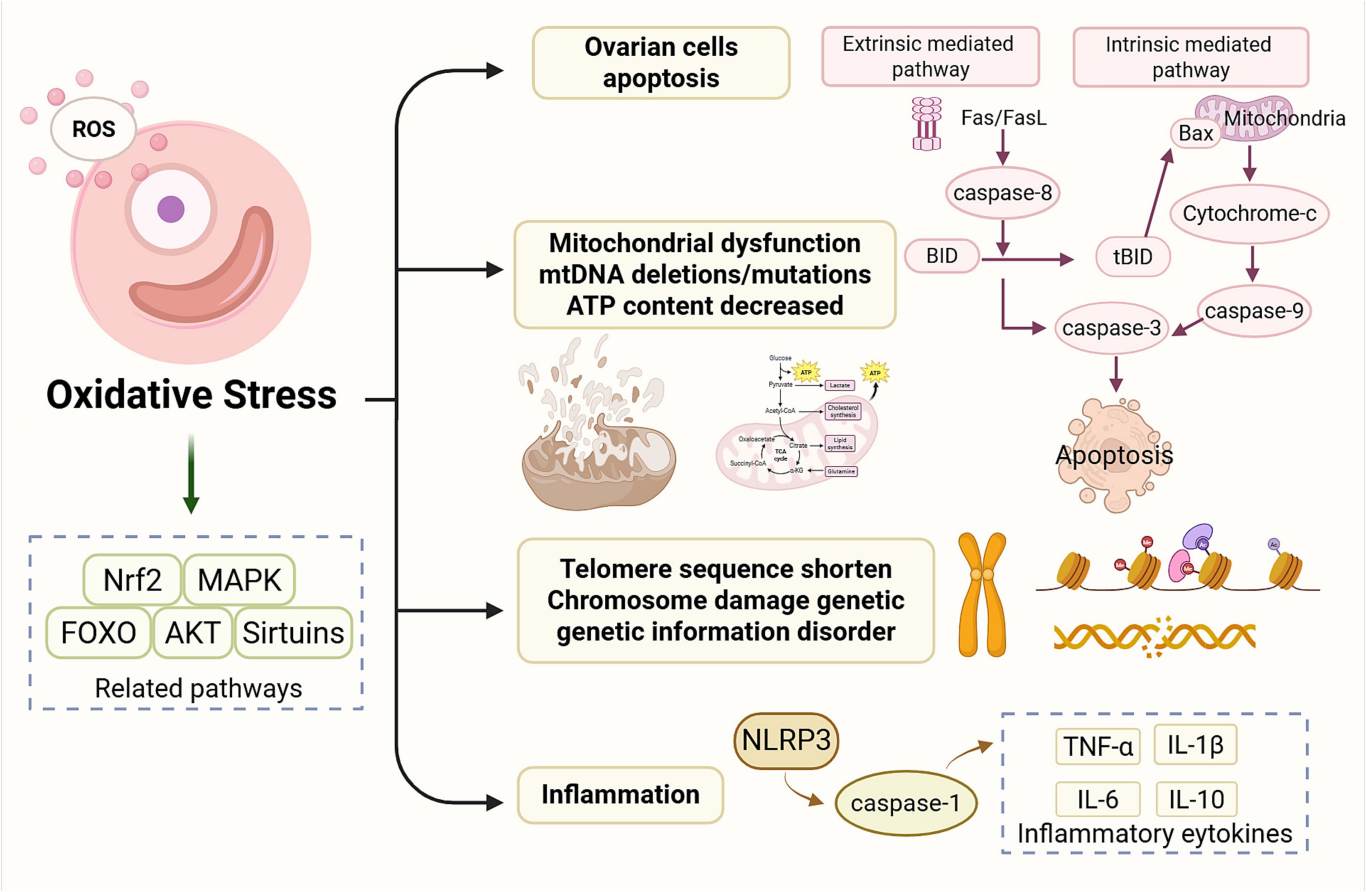
Oxidative stress-induced cellular processes contributing to ovarian aging. ROS accumulation triggers oxidative stress and activates multiple signaling pathways, including Nrf2, MAPK, FOXO, AKT, and sirtuins. Excessive oxidative stress promotes apoptosis of ovarian cells through both extrinsic and intrinsic mitochondrial pathways, converging on cell death. Oxidative damage also impairs mitochondrial integrity, leading to mtDNA deletions or mutations, reduced ATP production, and loss of mitochondrial membrane stability. Oxidative stress accelerates telomere shortening, induces chromosomal instability, and disrupts genomic integrity, collectively impairing ovarian function. In addition, ROS activates inflammasome signaling, leading to increased secretion of pro-inflammatory cytokines alongside dysregulation of anti-inflammatory mediators. ROS, reactive oxygen species; Nrf2, nuclear factor erythroid 2–related factor 2; MAPK, mitogen-activated protein kinase; FOXO, forkhead box O; AKT, protein kinase B; mtDNA, mitochondrial DNA; ATP, adenosine triphosphate; Fas, Fas cell surface death receptor; FasL, Fas ligand; BID, BH3-interacting domain death agonist; tBID, truncated BID; Bax, BCL2-associated X protein; NLRP3, NOD-like receptor family pyrin domain containing 3; TNF-α, tumor necrosis factor alpha; IL-1β, interleukin-1 beta; IL-6, interleukin-6; IL-10, interleukin-10. Created in BioRender. Chen, J. (2026) https://BioRender.com/ymfm5nr.

The free radical theory proposed in the 1950s is regarded as the classical theoretical foundation for ovarian aging. Free radicals are highly reactive oxidative products, encompassing reactive oxygen species (ROS) and reactive nitrogen species (RNS). Oxidative stress is a core mechanism underlying the occurrence and development of ovarian aging, with the key factor being the abnormal accumulation of ROS in ovarian tissues (24), a process that directly impacts the physiological functions of follicular development, angiogenesis, and sex hormone synthesis (25). Once the level of ROS is imbalanced, the excessive oxygen radicals trigger lipid peroxidation, which contributes to the increase of malondialdehyde (MDA) and decreased superoxide dismutase (SOD) activity, resulting in cell membrane damage and ovarian tissue dysfunction, thereby accelerating ovarian aging (26). Increased protein and lipid damage induced by reactive oxygen species in primordial follicles of advanced maternal age (27). Oxidative stress not only directly damages cell structures, but also mediates ovarian aging through mitochondrial dysfunction, chronic inflammation activation, apoptosis induction, and telomere shortening acceleration (28). This will be discussed further below (Figure 1). Moreover, high levels of ROS activate the signaling pathways, such as phosphatidylinositol 3-kinase (PI3K)/protein kinase B (Akt), mitogen-activated protein kinases (MAPK), and Kelch-like ECH-Associating protein 1/nuclear factor erythroid 2 related factor 2 (Nrf2)/antioxidant response element (ARE), which further exacerbate follicular atresia and functional failure (29).

### 2.2 Chronic inflammation

Chronic inflammation is widely recognized as a key driving force in ovarian aging (30). During the aging process, inflammatory signaling pathways are persistently upregulated in the ovary, as evidenced by elevated levels of pro-inflammatory cytokines and chemokines, together with enhanced lymphocyte-mediated immune responses. These alterations reflect a progressive remodeling of the intraovarian immune microenvironment and the establishment of a chronic low-grade inflammatory state. With advancing age, immune tolerance gradually declines, while danger signals such as ROS accumulation and DNA damage increase within ovarian tissue. These stimuli promote the assembly of the NOD-like receptor protein 3 (NLRP3) inflammasome in innate immune cells through its association with apoptosis-associated speck-like protein containing a CARD and caspase-1, thereby triggering downstream inflammatory cascades. Activation of this pathway not only suppresses autophagic capacity and enhances apoptotic signaling, but also disrupts the local homeostatic environment required for normal follicular development. Consequently, oocyte quality is compromised, follicular growth and ovulatory function are impaired, and depletion of the ovarian reserve is accelerated (31–33). Meanwhile, sustained NLRP3 inflammasome activation induces the cascading release of inflammatory cytokines such as interleukin-1beta (IL-1β) and IL-18 (34, 35). Among these, pro-inflammatory factors such as IL-1*α*, IL-1β, tumor necrosis factor-alpha (TNF-α) and IL-6 are particularly critical, especially TNF-α, which can directly induce follicular apoptosis and accelerate ovarian function decline (32, 36, 37).

Immune cells, especially macrophages, play indispensable roles in follicular growth, follicular atresia, ovulation, and corpus luteum formation and regression (38). Accumulating evidence indicates that the number of multinucleated giant cells increases markedly in the aging ovary, suggesting age-associated alterations in macrophage phenotype and function (39, 40). It has been proposed that ovarian aging promotes a shift of macrophages toward an alternatively activated phenotype, contributing to multinucleated giant cells formation and facilitating the abnormal accumulation of specific immune cell populations, particularly lymphocytes (41, 42). Such remodeling of immune cell composition and function may further amplify local inflammatory signaling, aggravate ovarian tissue damage, and ultimately accelerate the progression of ovarian aging.

### 2.3 Dysregulated apoptosis and autophagy

Excessive apoptosis and dysregulated autophagy in ovarian granulosa cells (GCs) and oocytes constitute key mechanisms underlying follicular atresia and ovarian aging (43). Disruption of autophagy homeostasis impairs cellular clearance of senescent and misfolded proteins (44). Reduced expression of autophagy-related proteins (e.g., ATG5 and ATG12) in rats, coupled with impaired autophagy function in human granulosa cells (hGCs) from advanced maternal age women, leads to diminished granulosa cell numbers, disrupted follicular development, and associated metabolic abnormalities, thereby accelerating ovarian aging (45). ROS may serve as a potential bridging factor linking autophagy to ovarian aging. ROS promote the formation of non-degradable lipofuscin within lysosomes, and these cross-linked protein residues accumulate with age, leading to reduced lysosomal degradative efficiency and, consequently, impaired oocyte quality (46). Apoptosis within the ovary exhibits unique regulatory mechanisms and factors, influenced by the hypothalamic–pituitary-ovarian axis and hormones such as follicle-stimulating hormone (FSH), luteinizing hormone (LH), progesterone (P), and especially estradiol (E_2_) (47, 48). However, it is primarily initiated through either the extrinsic death receptor pathway or the intrinsic mitochondrial-mediated pathway (12).

The extrinsic pathway utilizes death receptors and their corresponding ligands to induce apoptosis in granulosa cells and oocytes, including TNF-*α*/TNF receptor, Fas/FasL, and tumor necrosis factor-related apoptosis-inducing ligand (TRAIL)/TRAIL receptor (49, 50). Binding of death receptor ligands recruits the adaptor molecule caspase-8, leading to its dimerisation and activation. This subsequently induces apoptosis via two pathways: direct cleavage and activation of caspase-3 and caspase-7, or truncated BID, which links to the mitochondrial-mediated intrinsic apoptosis pathway (51). Intrinsic apoptotic pathways involving mitochondria, such as DNA damage or endoplasmic reticulum stress, can induce or activate BH3-only proteins, leading to their oligomerization on the mitochondrial outer membrane and the formation of pores, ultimately causing mitochondrial outer membrane permeabilization (MOMP) (52). Following MOMP, multiple intermembrane space proteins, such as cytochrome c, are released from the mitochondrial intermembrane space, which in turn activates caspase-9 and caspase-3, ultimately leading to cell death (53, 54). Members of the Bcl-2 protein family are involved in regulating this pathway, including anti-apoptotic proteins (Bcl-2) and pro-apoptotic proteins (Bax). Compared with normal follicles, aged rat ovaries exhibit a markedly increased number of atretic follicles, accompanied by reduced gene and protein expression of Bcl-2 and elevated expression of Bax (55). The anti-apoptotic protein Bcl-2 prevents MOMP by binding to BH3-only domain proteins and activated Bax.

### 2.4 Mitochondrial dysfunction

**Figure 2:**
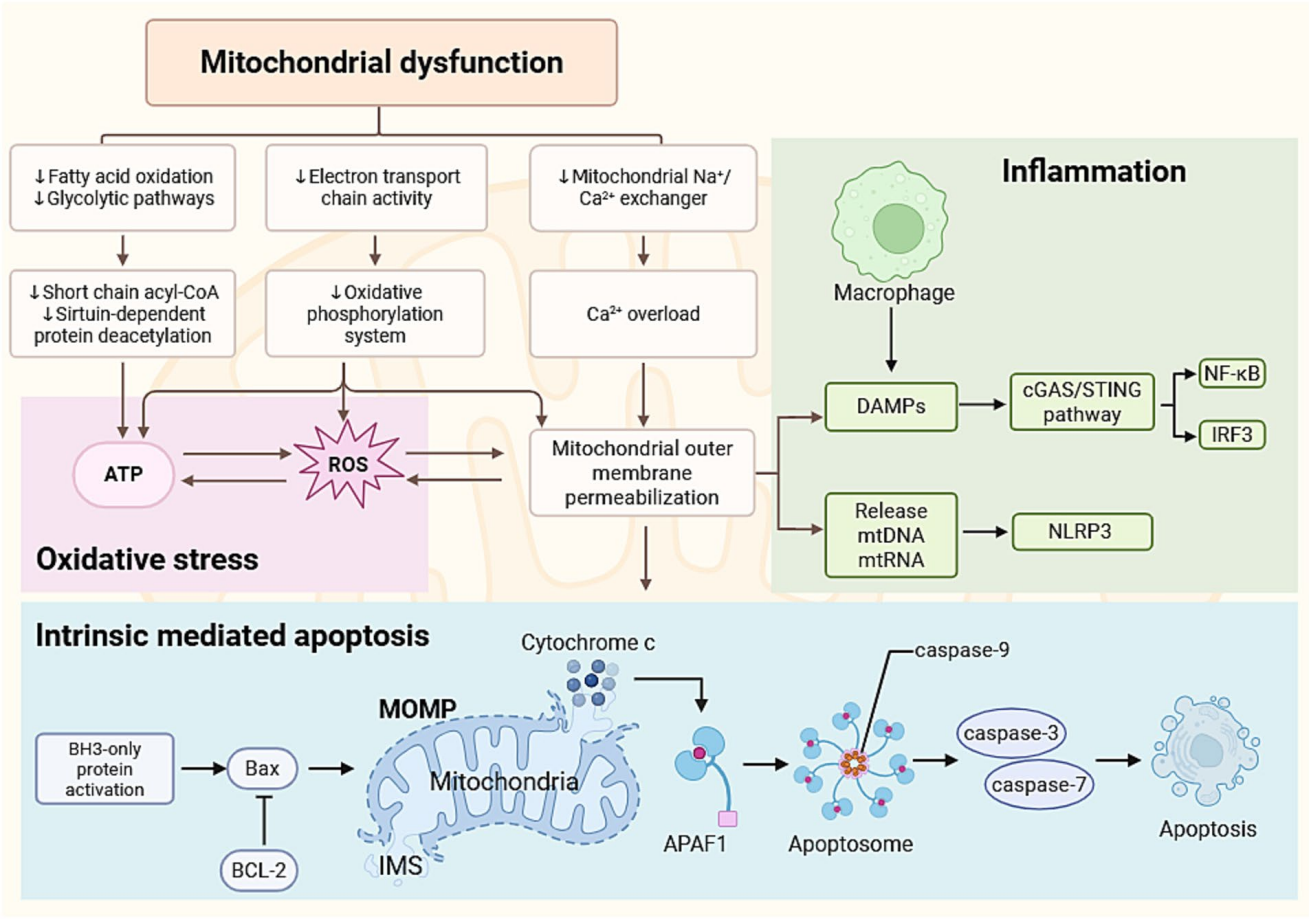
Mitochondrial dysfunction driven oxidative stress, inflammation, and intrinsic apoptosis in ovarian aging. Mitochondrial dysfunction reduces cellular energy production and disrupts metabolic homeostasis, leading to oxidative stress. Excessive oxidative stress impairs mitochondrial membrane integrity and promotes intrinsic apoptosis through cytochrome-c release and caspase activation. Damaged mitochondria also release mtDNA and other DAMPs, which activate inflammatory pathways such as cGAS–STING, NF-κB, and the NLRP3 inflammasome. ATP, adenosine triphosphate; ROS, reactive oxygen species; DAMPs, damage-associated molecular patterns; mtDNA, mitochondrial DNA; mtRNA, mitochondrial RNA; NLRP3, NOD-like receptor family pyrin domain containing 3; cGAS, cyclic GMP–AMP synthase; STING, stimulator of interferon genes; NF-κB, nuclear factor kappa B; IRF3, interferon regulatory factor 3; MOMP, mitochondrial outer membrane permeabilization; Bax, BCL2-associated X protein; BCL-2, B-cell lymphoma 2; IMS, intermembrane space; APAF1, apoptotic protease activating factor 1. Created in BioRender. Chen, J. (2026) https://BioRender.com/bib7ajx.

Mitochondria are central to energy metabolism in ovarian cells and are essential for maintaining oocyte function (56). The mechanisms by which mitochondrial dysfunction drives ovarian aging include impaired mitochondrial fusion and fission, reduced membrane potential (MMP), mitochondrial DNA (mtDNA) mutations, altered ATP metabolism, and defects in electron transport chain function (57). This leads to dysregulation of cell signaling and inefficient energy production in the ovary (58, 59), affecting critical processes such as rupture of germinal vesicles and microtubule assembly for meiosis (60, 61), thereby accelerating ovarian aging. Moreover, mitochondrial dysfunction readily interacts with mechanisms such as oxidative stress, inflammation, and apoptosis, accelerating primordial follicle depletion and precipitating reproductive potential loss (Figure 2). The ovarian intrinsic apoptosis pathway involving mitochondria and Bcl-2 family proteins has been discussed previously. Oxidative stress induces mtDNA mutations, mediates abnormal mtDNA-protein cross-linking, and impairs oxidative phosphorylation and ATP synthesis, thereby causing mitochondrial dysfunction through multiple pathways (62). Mitochondrial dysfunction further exacerbates ROS leakage from the electron transport chain, intensifying intracellular oxidative stress damage (57). This cascade of damage accelerates ovarian aging processes. Mitochondrial dysfunction may also activate innate immune pathways, such as NLRP3 inflammasome and NF-κB signaling, through damage-associated molecular patterns, inducing persistent ovarian inflammation that contributes to oocyte degeneration and ovarian aging (63). Increased permeability of the mitochondrial outer membrane enhances mtDNA leakage into the cytoplasm, subsequently activating inflammatory pathways including cGAS-STING (64). The ovary relies primarily on macrophages for the recognition and clearance of cellular damage (65). Macrophage function is hormonally regulated; during ovarian aging, declining oestradiol levels diminish their anti-inflammatory capacity while enhancing pro-inflammatory activity, thereby promoting the accumulation of mitochondrial damage-associated molecular patterns (66, 67).

### 2.5 Genomic instability and epigenetic modifications

Genomic instability denotes the cumulative tendency for permanent, heritable alterations in genomic DNA sequences, encompassing point mutations, frameshift mutations, and chromosomal aberrations, manifesting as multi-level disruptions including DNA damage and telomere attrition (68). Oxidative stress, metabolic wastes and environmental toxins can lead to DNA damage, inducing DNA breaks, base oxidation in the GCs and reduced DNA repair, thereby reducing oocyte quality and promoting follicular atresia (28, 69, 70). Furthermore, DNA damage can accelerate primordial follicle depletion and contribute to the decline in reproductive function by activating phosphatase and tensin homolog (PTEN)/PI3K/Akt/mammalian target of rapamycin (mTOR) signaling pathways (69). Conversely, telomeres, located at the ends of chromosomes, play a crucial role in protecting chromosome integrity and maintaining genome stability, with their length regulated by telomerase activity (71, 72). The decrease in telomerase activity and telomere length in the ovary promote premature follicle loss and directly accelerates the aging of ovarian cells (73). Epigenetic modifications can regulate gene expression and biological properties by influencing chromatin conformation without altering DNA sequences (74). During female germ cell aging, multiple abnormal epigenetic modifications are frequently observed, including aberrant DNA methylation, imbalanced histone modifications, and disrupted non-coding RNA regulation (75). Methylation abnormalities accompanying ovarian aging promote ovarian cell apoptosis and follicular depletion (76). Histone modifications regulate target gene activation or suppression by modulating chromatin structure; their imbalance may disrupt oocyte maturation and impair female fertility (77). Alterations in non-coding RNAs may regulate post-transcriptional and translational mechanisms, thereby influencing follicular development, maturation, and ovarian aging (78). Importantly, genomic instability does not act in isolation; instead, it forms a self-reinforcing network with oxidative stress, mitochondrial dysfunction, inflammation, and apoptosis, collectively accelerating follicular atresia and irreversible depletion of the ovarian reserve.

Ovarian aging is a complex and multifactorial process involving coordinated alterations at physiological, molecular, and cellular levels, including oxidative stress, mitochondrial dysfunction, dysregulated apoptosis and autophagy, chronic inflammation, and genomic instability. These mechanisms form an integrated and highly interconnected network that collectively drives the progressive decline of ovarian structure and function. As these pathogenic processes accumulate, dysfunction of the hypothalamic–pituitary-ovarian axis emerges, accompanied by reduced ovarian responsiveness and widespread endocrine imbalance (79).

## 3 MFH species and active ingredients in ovarian aging interventions

**Figure 3:**
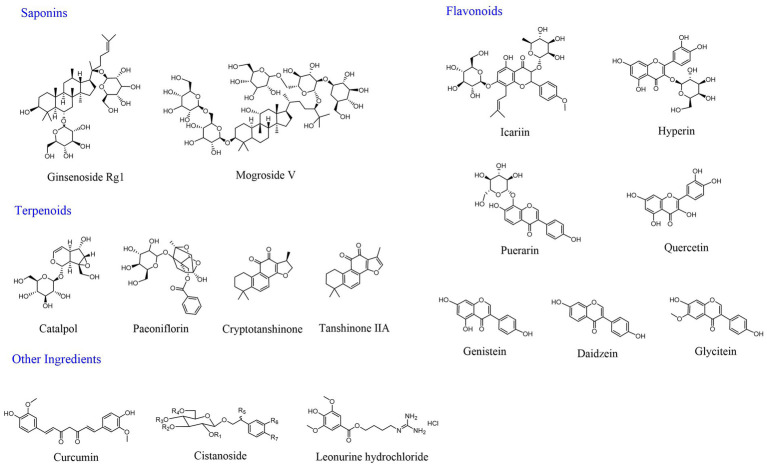
Structure of a representative active ingredient for improving ovarian aging.

**Figure 4:**
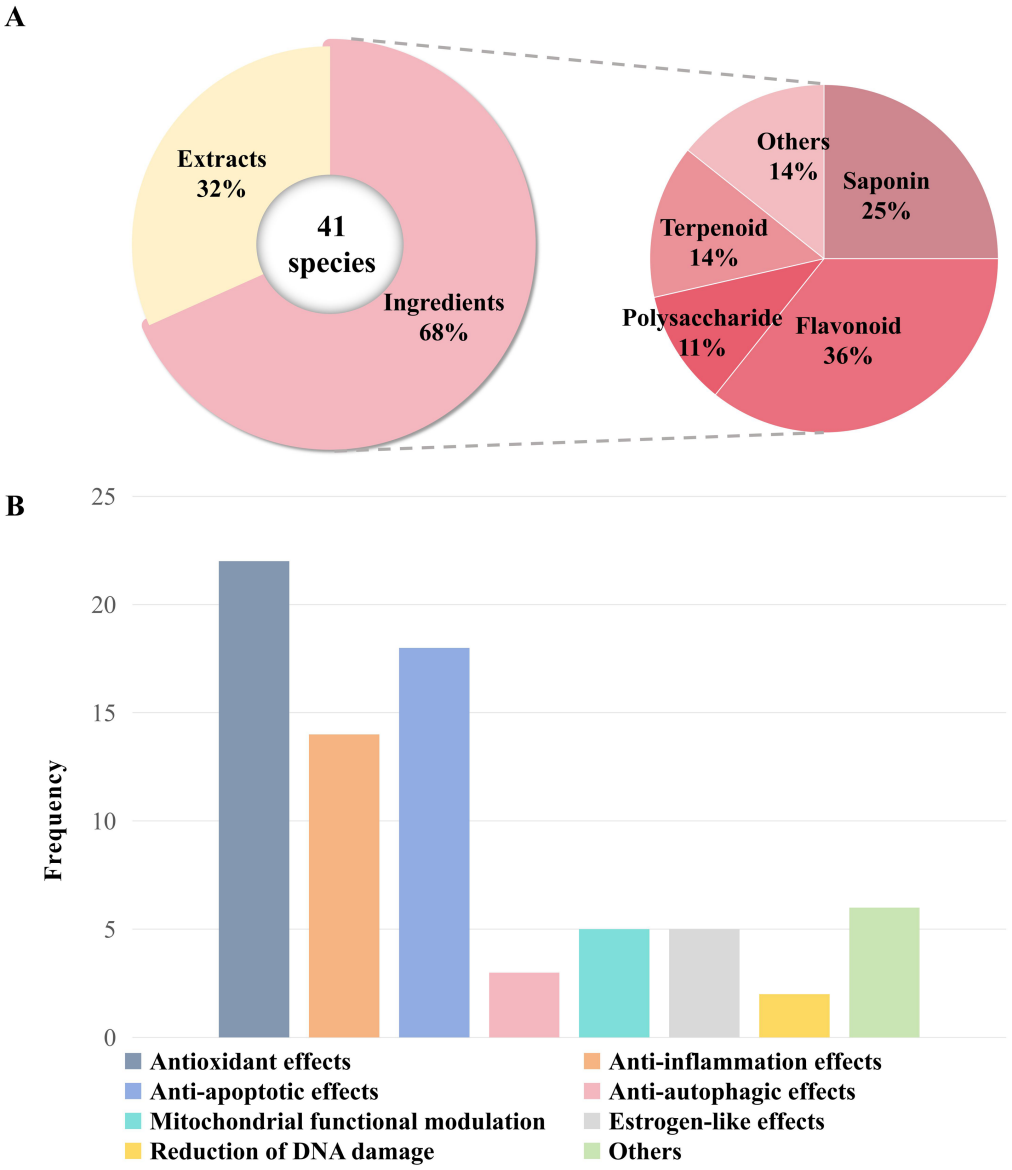
Analysis of MFH species and active ingredients showing ovarian anti-aging effects in this review. **(A)** Classification about MFH species and active ingredients. **(B)** The relational mechanism of MFH species and active ingredients on ovarian aging.

This review summarized the positive effects of MFH extracts and their active ingredients on ovarian aging, discussed their sources, potential effects and mechanisms of action (Tables 1–4), and presented the chemical structures of the representative active ingredients in Figure 3. ‘Herbal extracts’ refer to complexes processed by aqueous or alcoholic extraction, which usually contain saponins, flavonoids, polysaccharides, terpenoids and other active ingredients, demonstrating the overall efficacy and multi-targeting advantages of herbs. ‘Active ingredients’ are single compounds with defined pharmacological activities that are further isolated and purified from these extracts. Both have distinct focuses in the study and complement each other to support the intervention studies of MFH species on ovarian aging (Figure 4).

**Table 1:** The role and mechanism of MFH extract in ameliorating ovarian aging.

Extract of MFH	Experimental model	Dose and duration	Efficacy	Mechanism	Reference
*Panax ginseng* C. A. Mey (Red ginseng)	D-gal-induced POF mice	200, 400 mg/kg/day; 28 days	Estrous cycle recovery; ovarian index, follicle count↑; E_2_↑, FSH↓	Antioxidant, anti-apoptosis (Nrf2/HO-1 pathway, PI3K/Akt pathway↑)	(81)
*Panax ginseng* C. A. Mey (American ginseng)	VCD-induced POF mice	2.25 g/kg/day; 44 days	Ovarian index, E_2_↑, LH, FSH↓	Anti-inflammatory	(82)
	VCD-induced POF mice	1.125, 2.25 and 4.5 g/kg/day; 30 days	Ovarian morphology improved, E_2_↑, FSH↓	Anti-inflammatory, antioxidant, anti-apoptosis	(83)
*Lycium barbarum* L.	Naturally aged mice	6, 12 g/L/day; 30 days	Fertility and offspring quality↑; AMH, E_2_↑	Antioxidant, anti-inflammatory (Nrf2, PRDX4↑)	(84)
*Salvia miltiorrhiza* Bunge	CTX-induced ovarian injury in mice	0.02 g/0.15 mL·20 g·bw; 41 days	Ovarian index, follicle count↑, atretic follicle count↓, ovarian morphology improved; AMH↑	Antioxidant	(212)
*Rehmannia glutinosa* (Gaertn.) DC.	CTX-induced POI in mice	1,200, 4,800 mg/kg, q2d; 12 weeks	Follicle count↑; E_2_↑, FSH↓	Anti-inflammatory, anti-apoptosis (AMPK/mTOR↑ pathway)	(85)
*Cistanche deserticola* Y. C. Ma	Cisplatin-induced POF mice	5, 10 g/kg/day; 2 weeks	Follicle count↑, apoptotic cell count↓, ovarian morphology improved; E_2_↑, FSH↓	Anti-apoptosis, improving mitochondrial function	(86)
	TGs-induced POF in rats	5, 10 and 20 g/kg/day; 20 days	E_2_, AMH↑, FSH↓	Anti-inflammatory, anti-apoptosis	(213)
*Angelica sinensis* (Oliv.) Diels	TGs-induced POI in rats	25, 100 mg/kg/day; 15 days	Follicle count↑, atretic follicle count↓; E_2_, AMH↑, FSH, LH↓	Anti-inflammatory (Nrf2/HO-1 pathway↑)	(88)
*Paeonia lactiflora* Pall.	Naturally aged mice	26.5, 53 mg/kg/day; 4 weeks	Follicle count and fertility↑	Improving ovarian microcirculation	(90)
*Ligustrum lucidum* W. T. Aiton	TGs-induced POI in rats	1.6 g/kg/day; 21 days	Follicle count↑, atretic follicle count↓, ovarian morphology improved; P, E_2_↑, LH, FSH↓	Anti-apoptosis	(214)
*Portulaca oleracea* L.	D-gal-induced aging mice	200 mg/kg/day; 21 days	LH, FSH↓, E_2_, P↑	Antioxidant	(215)
Pearl	TGs-induced POF in rats	185, 370, and 740 mg/kg/day; 30 days	Follicle count↑, atretic follicle count↓, ovarian morphology improved; E_2_, AMH↑, FSH, LH↓	Antioxidant, anti-apoptosis and activating autophagy (MAPK pathway↓)	(216)
*Dendrobium nobile* Lindl.	D-gal-induced aging mice	200 mg/kg; 8 weeks	Ovarian morphology improved	Remodeling the gut micro-ecosystem, antioxidant	(165)
*Asparagus cochinchinensis* (Lour.) Merr.	Naturally aged rats	12 g/L; 72 weeks	Atretic follicle count↓, luteal degeneration mitigation	/	(217)

↑: activation or upregulation; ↓: inhibition or downregulation.

**Table 2:** The role and mechanism of saponin in MFH in improving ovarian aging.

Source	Ingredients	Experimental model	Dose and duration	Efficacy	Mechanism	Reference
*Panax ginseng* C. A. Mey. (Red ginseng)	Heat-transformed saponin	CTX-induced POF model rats, KGN cells	150, 300, and 600 mg/kg/d; Time unknown (*in vivo*) 5, 10, and 20 mg/L; 24 h (*in vitro*)	Follicle count↑, atretic follicle count↓, KGN cells viability↑, apoptosis rate↓; E_2_, AMH↑, FSH↓	Anti-inflammatory, antioxidant (p38 MAPK/NF-κB p65 pathway↓)	(100)
*Panax ginseng* C. A. Mey.	Ginsenoside Rg1	D-gal-induced POI in mice	20 mg/kg/day; 28 days	Ovarian morphology improved, follicle count, CL count↑, E2, AMH↑, FSH↓	Anti-inflammatory, antioxidant (p21-p53-STK pathway↑)	(99)
		D-gal-induced POF mice	20 mg/kg/day; 28 days	Follicle count, CL count↑, ovarian morphology improved, fertility↑; E_2_, AMH↑, FSH↓	Antioxidant, anti-inflammatory	(98)
		Naturally aged mice, human GCs, KGN cells	10 mg/kg; 2 weeks (*in vivo*) 1 mol/L; 10 h (*in vitro*)	Fertility↑, KGN cells apoptosis rate↓	Antioxidant	(101)
		Naturally aged mice	6 mg/kg/3 day; 6–7 months	Estrous cycle recovery	Antioxidant, anti-inflammatory	(218)
		D-gal-induced POF mice	20 mg/kg/day; 28 days	Weight growth rate, ovarian weight coefficient, follicle count↑; E_2_, LH↑, FSH↓	Antioxidant	(102, 103)
		D-gal-induced POF mice	20 mg/kg/day; 28 days	Estrous cycle recovery, the positive rate of ovarian SA-β-Gal staining↓	Activating autophagy (PI3K/Akt/mTOR pathway↑)	(104)
		GCs of cisplatin-induced POF rats	100, 200, 300 ng/mL; 24 h,48 h	Cell proliferation rate↑	Anti-apoptosis (FSHR/PI3K/Akt pathway↑)	(219)
		Radiation-induced POI rats	20 mg/kg/day; 4 weeks	Ovarian index, follicle count↑, ovarian morphology improved; E_2_↑, FSH↓; TUNEL positive cell count↓	Improving mitochondrial function	(105)
*Siraitia grosvenorii* (Swingle.) C. Jeffrey ex A. M. Lu et Z. Y. Zhang	Mogroside V	Porcine oocyte	25, 50, and 100 μM; 24 h	Activation rate↑, apoptosis rate↓; blastocyst formation rate↑	Antioxidant, maintaining genome stability, improving mitochondrial function	(114)
	Mogroside	Naturally aged mice	600 mg/kg/day; 34 weeks	Estrous cycle recovery, follicle count↑	Anti-inflammatory	(115)
*Astragalus membranaceus* (Fisch.) Bge.var.mongholicus (Bge.) Hsiao	Astragaloside IV	CTX-induced POI in rats	40 mg/kg/day; 24 days	Ovarian morphology improved, apoptotic cell count↓; AMH, E_2_↑, FSH↓	Antioxidant, anti-inflammatory, anti-apoptosis	(220)
	Astragaloside	CTX-induced POI in rats	20, 40 and 80 mg/kg/d; 28 days	Follicle count↑; E_2_, AMH↑, FSH, LH↓	Anti-inflammatory (NF-κB pathway↓)	(221)
*Dioscorea oppositifolia* L.	Diosgenin	Naturally aged mice	200 mg/kg/day; 3 months	Follicle count, fertility↑; AMH↑	/	(222)

↑: activation or upregulation; ↓: inhibition or downregulation.

**Table 3:** The role and mechanism of flavonoid in MFH in improving ovarian aging.

Source	Ingredients	Experimental model	Dose and duration	Efficacy	Mechanism	Reference
*Epimedium acuminatum* Franch.	Icariin	D-gal-induced ovarian aging mice	50, 100, and 200 mg/kg/day; 30 days	Follicle count, fertility↑, atretic follicle count↓; E_2_, AMH↑, FSH, LH↓; optimal concentration 100 mg/kg	Anti-apoptosis	(125)
		Cisplatin-induced POF mice, KGN cells	30 mg/kg/day; 21 days (*in vivo*) 5 μg/mL; 6 h (*in vitro*)	Estrous cycle recovery, follicle count↑; KGN cells apoptosis rate↓	Antioxidant, anti-apoptosis (Nrf2/ARE pathway↑)	(126)
		CTX-induced POI rats	15, 30, and 60 mg/kg/day; 28 days	Ovarian index, follicle count↑, atretic follicle count↓, ovarian morphology improved; E_2_, AMH↑, FSH↓	Antioxidant, anti-apoptosis (PI3K/Akt/mTOR pathway↑)	(127)
		Porcine oocyte	5, 50, or 500 μM; 24 h	Blastocyst development rate↑	Maintaining genome stability, antioxidant, anti-apoptosis, estrogen-like effects	(123, 124)
		Pzp3-induced autoimmune POI mice	40 mg/kg/day; 28 days	Ovarian morphology improved, follicle count↑; FSH, LH, AZPAb↓, AMH↑	Modulating immunity (Nrf2/HO-1/SIRT1 pathway↑)	(128)
		D-gal-induced POF mice, GCs	10, 50, and 100 mg/kg/day; 42 days (*in vivo*) 100 nM, 1 and 10 μM; 6 h (*in vitro*)	Follicle count, fertility, GC viability↑; FSH, LH↓, E_2_, AMH↑	Promoting DNA damage repair	(129)
*Crataegus pinnatifida* Bunge, etc.	Hyperin	TG-induced POI mice	75 mg/kg/day; 28 days	Follicle count, CL count↑, apoptotic cell count↓, ovarian morphology improved; E_2_, AMH↑, FSH↓	Antioxidant, anti-apoptosis (Nrf2/HO-1 pathway, PI3K/Akt pathway↑)	(133)
		Triptolide-induced KGN cells	1, 10, and 50 μg/mL; 24 h	Survival rate↑, apoptosis rate↓; P, E_2_↑	Anti-apoptosis (Akt/TSC1/mTORC1 pathway↑)	(134)
*Pueraria lobata* (Willd.) Ohwi	Puerarin	CTX-induced POF mice	100, 200 mg/kg/day; 28 days	Follicle count↑, atretic rate↓	Anti-apoptosis, antioxidant (Wnt/β-catenin pathway↑)	(141)
		VCD-induced DOR rats	50, 100, and 300 mg/kg/day; 45 days	Follicle count↑, apoptotic cell count↓, ovarian morphology improved; FSH, LH↓, E_2_↑	Anti-apoptosis	(142)
*Glycine max* (L.) Merr.	Soy isoflavones	Naturally aged rats	150 mg/kg/day; 8 weeks	Atretic follicle count↓	Anti-apoptosis, antioxidant	(147)
		Naturally aged menopausal rats, rat GCs	50, 158, and 500 mg/kg/day; 8 weeks (*in vivo*) 0.1, 1, 5, 10, and 100 μ mol/L; 48 h (*in vitro*)	/	Estrogen-like effects	(149)
	Genistein	Naturally aged mice	160 mg/kg/day; 4 months	Follicle count↑; delayed cessation of the estrous cycle	/	(150)
		Naturally aged rats	160 mg/kg/day; 4 months	Follicle count↑, atretic follicle count↓	/	(151)
*Alpinia galanga* (L.) Willd., etc.	Quercetin	CTX-induced POI mice	12.5, 25 and 50 mg/kg/day; 4 weeks	Ovarian morphology improved; FSH, LH↓, AMH, E_2_, P↑	Improving mitochondrial function, inhibiting pyroptosis (PGC1-α pathway↑)	(153)
		KGN cells	40 μmol/L; 48 h	Tunel-positive cells↓	Anti-apoptosis (JAK2/STAT3 pathway↑)	(154)
		Naturally aged menopausal rats, GCs of H_2_O_2_-induced rat	12.5, 25 and 50 mg/kg/day; 90 days (*in vivo*) 5, 20, and 50 μM_;_ 6 h (*in vitro*)	GC viability↑	Antioxidant	(155)
		CTX-induced POI rats	25, 100 mg/kg/day; 14 days	Ovarian index, follicle count↑, atretic follicle count↓; FSH↓, E_2_, AMH↑	Anti-inflammatory (SDF-1/CXCR4 pathway↓)	(156)
		KGN cells	25 μM; 96 h	Cell count↑, apoptotic cell count↓; E_2_↑	Promoting estrogen synthesis (CYP19A1↑)	(223)
		TG-induced POF rats	600 mg/kg/day; 30 days	Ovarian index, follicle count↑, atretic follicle count↓; AMH, E_2_↑, FSH, LH, FSH/LH↓	/	(157)
*Astragalus cuscutae* Bunge	Total flavonoids	TG-induced POF rats	530.1 mg/kg/day; 30 days	Ovarian index, follicle count↑, atretic follicle count↓; AMH, E_2_↑, FSH, LH, FSH/LH↓	/	(157)
*Rubus chingii* Hu	Isoquercitrin	AAPH-induced KGN cells	100 μM; 24 h	Cell viability↑	Antioxidant	(159)
*Morus alba* L.	Astragalin	Naturally aged rats, CdCl2-induced GCs	3, 15, and 30 mg/kg/day; 2 weeks (*in vivo*) 0.05, 0.1, 0.25, and 0.5 mM; 24 h (*in vitro*)	GC proliferation rate↑, apoptosis rate↓; E_2_, P_4_↑, FSH, LH↓	Anti-apoptosis	(224)
*Astragalus membranaceus* (Fisch.) Bunge, etc. (225)	Kaempferol	AR-DOR mice	100 mg/kg/day; 4 weeks	AMH, E_2_↑, FSH↓	Antioxidant	(226)

↑: activation or upregulation; ↓: inhibition or downregulation.

**Table 4:** The role and mechanism of other active ingredients in MFH in improving ovarian aging.

Classification	Source	Ingredients	Experimental model	Dose and duration	Efficacy	Mechanism	Reference
Polysaccharide	*Dendrobium nobile* Lindl.	*Dendrobium* polysaccharides	Naturally aged mice	70 mg/kg/day; 10 weeks	Follicle count↑, ovarian morphology improved; E_2_↑	Antioxidant, anti-inflammatory, improving mitochondrial function (NF-κB pathway, p53/Bcl-2 pathway↓)	(166)
	*Lycium barbarum* L.	*Lycium barbarum* polysaccharides	Naturally aged rats	20, 40, and 60 mg/kg/day; 30 days	Estrogen, progesterone, IGF-I↑, IGFBP-1↓	/	(227)
			D-gal-induced POI in mice	160, 520 mg/kg/day; 12 weeks	Follicle count, fertility↑; FSH, LH↓	Remodeling the intestinal micro-ecosystem	(169)
			D-gal-induced POF mice	60 mg/kg/day; 28 days	Follicle count↑, ovarian morphology improved; E_2_, LH↑, FSH↓	Activating autophagy (AMPK/SIRT1 pathway↑)	(170)
	*Angelica sinensis* (Oliv.) Diels	*Angelica sinensis* polysaccharides	Pzp3-induced autoimmune POF mice	100, 200, and 400 mg/kg/day; 4 weeks	Ovarian index↑	Antioxidant (Akt/FOXO3 pathway↑)	(228)
Terpenoid	*Rehmannia glutinosa* (Gaertn.) DC.	Catalpol	Naturally aged rats	1, 3, and 5 mg/kg/day; 4 weeks	Ovarian index↑, apoptotic cell count↓, ovarian morphology improved; E_2_, P_4_↑, FSH, LH↓	/	(177)
			GCs of cisplatin-induced rats	50, 100, and 200 μmol/L; 24 h	Cell viability, proliferation↑, cell apoptosis rate↓	Anti-apoptosis	(229)
			Tripterygium-induced POI rats	30, 60 mg/kg/day; 4 weeks	Ovarian index, follicle count↑, atretic follicle count, cell apoptosis rate↓, ovarian morphology improved; E_2_↑, FSH, LH↓	Antioxidant, anti-apoptosis (Hedgehog pathway↑)	(178)
	*Paeonia lactiflora* Pall.	Paeoniflorin	Cisplatin-induced DOR mice, cisplatin-induced KGN cells	75, 150 mg/kg/day; 4 weeks (*in vivo*) 1, 10, 20, and 50 μM; 48 h (*in vitro*)	Ovarian index, follicle count, CL count↑; E_2_, AMH↑, FSH↓	Restoration of E_2_ synthesis by GCs (FSHR/cAMP/PKA/CREB pathway↑)	(184)
	*Salvia miltiorrhiza* Bunge	Cryptotanshinone	CTX-induced POF mice	50, 100 mg/kg/day; 4 weeks	Ovarian morphology improved; AMH, E_2_↑, LH, FSH↓	Anti-apoptosis	(187)
			VCD-induced POI rats	50, 100 mg/kg/day; 4 weeks	Apoptotic cell count↓, ovarian morphology improved; E_2_↑, FSH, LH↓	Anti-apoptosis, antioxidant (SDF-1/CXCR4 pathway↑)	(188)
		Tanshinone IIA	Naturally aged mice	10, 20 and 40 μg/g/day; 2 weeks	Follicle count↑; AMH, E_2_↑, FSH, LH↓	Antioxidant	(189)
Polyphenol	*Curcuma longa* L.	Curcumin	Naturally aged mice	100 mg/kg/day; 28 days	Follicle count↑; FSH↓, AMH, E_2_↑	Anti-inflammatory (PTEN/Akt/FOXO3a pathway↓)	(195)
			FSH-R haploinsufficient Mice	25 mg/kg/day; 40 days	Follicle count↑, improved cell morphology	Anti-androgenic effect	(196)
			D-gal-induced POF mice	100 mg/kg/day; 42 days	Follicle count↑, apoptotic cell count↓; FSH, LH↓, E_2_, P, AMH↑	Antioxidant, anti-apoptosis (Nrf2/HO-1 pathway, PI3K/Akt pathway↑)	(197)
			Naturally aged mice	100 mg/kg/day; 33 weeks	Follicle count, fertility↑; AMH, E_2_↑, FSH↓	Antioxidant, anti-apoptosis	(198)
	*Cistanche deserticola* Y. C. Ma	Phenylethanoid glycosides	Menopausal syndrome model mice	50, 100, and 200 mg/kg/day; 21 days	E_2_, T↑, LH, FSH↓	/	(230)
			Perimenopausal rats	33.33, 66.67 and 133.33 mg/kg/day; 30 days	E_2_, T, BGP, β-EP↑; FSH, LH, GnRH↓	Estrogen-like effects	(231)
Alkaloid	*Leonurus japonicus* Houtt.	Leonurine hydrochloride	CTX-induced POI in rats	7.5, 15, and 30 mg/kg/day; 28 days	Follicle count, fertility↑, atretic follicle count↓; AMH, E_2_↑, FSH↓	Inhibiting pyroptosis (NLRP3/GSDMD pathway↓)	(207)
			CTX-induced mice of ovarian function decline, MCF-7 cells, MDA-MB-231 cells	0.65, 1.3, and 2.6 mg/kg/day; 7 days (*in vivo*) 3.3, 3.3 × 0.1, 3.3 × 0.01, 3.3 × 0.001 mmol/L; 72 h (*in vitro*)	Cell proliferation ↑, motility cycle restored, follicle count↑	Estrogen-like effects (ERα, ERβ↑)	(208)
Protein	*Dioscorea polystachya* Turcz.	Osteogenic Yam Protein	Naturally aged rats, rat GCs	2.5, 5, and 10 mg/kg/day; 6 weeks (*in vivo*) 1, 10, 100 nM; 12 h (*in vitro*)	FSHR, ovine aromatase↑, E_2_, P↑	/	(232)

↑: activation or upregulation; ↓: inhibition or downregulation.

### 3.1 Herbal extracts

*Panax ginseng* C. A. Mey, a perennial herb of the Araliaceae family, is widely used in traditional Chinese medicine and has significant ovarian anti-aging potential (80). Shang et al. (81) found that red ginseng extract effectively improved ovarian function in D-gal-induced POF mice, significantly increased the number of follicles at all levels, elevated E_2_ and anti-mullerian hormone (AMH) levels, and decreased FSH levels and senescent protein p53, p21 and p16. The mechanism underlying these effects was closely related to the activation of Nrf2/heme oxygenase-1 (HO-1) and PI3K/Akt signaling pathways, which exerted antioxidant and anti-apoptotic effects. Ge et al. (82, 83) reported that treatment of 4-vinylcyclohexene dioxide (VCD)-induced mice with American ginseng extract reduced the levels of prostaglandin E_2_, FSH and LH, and normalized E_2_ secretion. These changes significantly mitigated the ovarian pathological damage and abnormal ovulation, thereby enhancing fertility. The aqueous extract of American ginseng increased *microRNA-144* and *microRNA-29a* expressions, downregulated *PLA2G4A* mRNA and protein, regulated prostaglandin biosynthesis, and protected ovarian function. Meanwhile, American ginseng extract increased the expression levels of pregnancy-associated plasma protein A (PAPPA), stanniocalcin-2 (STC2), C-C motif chemokine 2 (CCL2), and NEL-like protein 1 (NELL1), which may improve the symptoms of POF by enhancing anti-inflammatory, antioxidant, and anti-apoptotic effects (83).

The MFH species, such as *Lycium barbarum* L., *Rehmannia glutinosa* (Gaertn.) DC., *Cistanche deserticola* Y. C. Ma, *Angelica sinensis* (Oliv.) Diels, and *Paeonia lactiflora* Pall., are commonly used in traditional Chinese medicine to regulate women’s reproductive health and have been shown to slow ovarian aging. Jiang et al. (84) supplemented aged female mice with *Lycium barbarum* berry extract, which significantly elevated fertility as well as AMH and E_2_ levels and attenuated the levels of oxidative damage markers 8-hydroxy-2′-deoxyguanosine (8-OHdG), gamma-phosphorylated histone H2AX (γH2AX), and the inflammatory factor IL-6. This effect was related to its activation of the antioxidant response via NRF2 and peroxiredoxin 4. Zhang et al. (85) reported that treatment with *Rehmannia glutinosa* extract in cyclophosphamide (CTX)-induced POI mice resulted in an increased follicle number, a significant increase in E_2_ and a significant decrease in FSH. These effects were mediated by the activation of AMP-activated protein kinase (AMPK)/mTOR signaling pathway to inhibit apoptosis. Pan et al. (86) found that *Cistanches herba* extract upregulated the level of mitofusin-2 (Mfn2) and the Bcl-2/Bax ratio, restored mitochondrial enzyme activity and MMP in ovarian tissues, and primarily mediated these effects through the inhibition of apoptosis and improvement of mitochondrial function to intervene in ovarian aging.

The aqueous extract of *Angelica sinensis* alleviated age-related physiological decline in mice (87). Ma et al. (88) established a rat model of POI induced by Tripterygium glycosides (TGs). After 15 days of administration of *Angelica sinensis* extract, serum E_2_ and AMH levels and follicle counts increased, while FSH, LH, and inflammatory factors TNF-*α*, IL-4, and IL-6 levels decreased, which was attributed to the activation of the Nrf2/HO-1 signaling pathway to exert anti-inflammatory effects. *Paeonia lactiflora* may exert anti-inflammatory and anti-apoptotic effects by mediating signaling pathways for the treatment of POI, including advanced glycation end products (AGE)/receptor for advanced glycation end products (RAGE), TNF, and IL-17 (89). Another study suggested that the aqueous extract of *Paeonia lactiflora* increased the expression of ovarian angiogenic factors vascular endothelial growth factor (VEGF) and visfatin, thereby restoring ovarian function by improving ovarian microcirculation in naturally aged mice (90).

### 3.2 Saponins

#### 3.2.1 Ginsenoside

Ginsenosides are a class of triterpenoid saponins with similar basic structures extracted from different parts of ginseng (91). As the major bioactive components in ginseng (81), ginsenosides exhibit a variety of biological activities, including lowering blood lipids and blood glucose, inhibiting inflammation and oxidative stress, inhibiting steatosis, protecting liver and cardiomyocytes, and possessing anti-aging and anti-tumor effects (92–97). Ginsenosides have significant potential to alleviate ovarian aging through antioxidant, anti-inflammatory, anti-apoptotic, and improved mitochondrial function.

He et al. (98, 99) used D-gal-induced ovarian functionally impaired mice as test subjects. After gavage treatment with ginsenoside Rg1, the pathological morphology of the ovary improved, the number of follicles increased, serum E_2_ and AMH levels increased, and FSH levels decreased. The mechanism underlying these effects involves the antioxidant and anti-inflammatory properties of ginsenoside Rg1 and the downregulation of the senescence signaling pathway p21-p53- serine/threonine kinase (STK). Meanwhile, Tao et al. (100) suggested that the anti-inflammatory and antioxidant effects of ginsenosides were through the regulation of the p38 MAPK/ nuclear factor kappaB (NF-κB) p65 signaling pathway. Zhou et al. (101) showed that treating ovarian granulosa cells from aged women and naturally aged mice with ginsenoside Rb1 decreased levels of lactate dehydrogenase (LDH), MDA, caspase-3 and caspase-9. Mechanistically, this effect was mediated by blocking the Akt/Forkhead box protein O1 (FoxO1) interaction, which inhibited the transcriptional regulation of apoptosis and, in turn, reduced age-related ovarian oxidative stress injury. Liu et al. (102–104) found that inhibiting silent information regulator sirtuin 1 (SIRT1) expression resulted in hypogonadism, delayed sexual maturation and infertility in mice. Ginsenoside Rg1 prevents POF in D-gal-induced mice by enhancing SIRT1 or inhibiting PI3K/Akt/mTOR autophagy signaling pathway. Zhu et al. (105) used ginsenoside Rg1 in combination with human amnion-derived mesenchymal stem cells transplantation to treat radiation-induced mouse models of POI, resulting in a significant increase in MMP and ATP production in oocytes. This suggests that the mechanism was related to improved mitochondrial function in oocytes.

#### 3.2.2 Mogroside

*Siraitia grosvenorii* (Swingle.) C. Jeffrey ex A. M. Lu et Z. Y. Zhang, a perennial vine in the Cucurbitaceae family, also known as Luo hanguo or monk’s fruit, has an important history in food and medicine (106). Triterpenoid saponins are the main bioactive components of *Siraitia grosvenorii*, with the highest content of mogroside V (107, 108). Modern pharmacological studies have shown that mogrosides exhibit immunomodulatory, anti-inflammatory, hypoglycemic, hypolipidemic, hepatoprotective, antioxidant, and anti-tumor effects (109–112).

In the reproductive system, mogroside attenuates oxidative stress damage, inflammatory damage and metabolic disorders, thereby improving oocyte quality. Mogroside restored meiotic defects and decreased oocyte quality in benzo(a)pyrene-exposed mice (113), and attenuated oocyte damage during aging *in vitro* (114). Aging oocytes, which are more likely to be activated due to reduced mass, decreased from 32.1 to 16.9%, while blastocyst formation increased from 9.5 to 16.0% after mogroside V treatment. Mogroside V reduced oxidative stress by upregulating SIRT1 expression, decreasing ROS levels, and reversing oocyte cytoskeletal abnormalities, mitochondrial dysfunction, and early apoptosis (114). After administration of mogroside in drinking water, the estrous cycle of naturally aged mice was restored and the number of follicles at all levels increased significantly, including a 2.8-fold increase in the number of sinus follicles. Mechanistically, the levels of inflammation-related genes (*Tnfα, Il6ra, Il10rb, Il2, Tgfb1, Lc3*) and TNF-α were significantly decreased in the aging ovaries, suggesting that the anti-aging effect may be partially attributed to the inhibition of ovarian inflammatory responses (115).

### 3.3 Flavonoids

#### 3.3.1 Icariin

*Epimedium acuminatum* Franch. is a traditional Chinese medicine that has been shown to strengthen the body, improve fertility, and relieve stress and fatigue (116). Icariin is the most abundant extracted component (117). This compound is a flavonoid glycoside with anti-inflammatory, anti-aging, and anti-tumor activities (118–122), and primarily intervenes in ovarian aging mainly through antioxidant, anti-apoptotic, estrogen receptor (ER) effects, immune modulation, and maintenance of genomic stability.

Icariin protected porcine oocytes from age-related damage in vitro, prevented oocyte skeleton abnormalities, and improves embryo developmental competence (123, 124). Wang et al. (125) found that icariin effectively improved ovarian function, fertility and E_2_ and AMH levels, and lowered FSH and LH levels in D-gal-induced ovarian aging mice, primarily through apoptosis inhibition. Li et al. (126) found that icariin improved ovarian function and morphology in cisplatin-induced POF mice, reduced ROS, MDA and Bax levels, competitively bound Keap-1 and activated the Nrf2/ARE pathway to inhibit oxidative stress, ferroptosis, and apoptosis. Another study suggested that icariin modulated the PI3K/Akt/mTOR pathway to achieve these effects (127). Chen et al. (128) showed that icariin had an immunomodulatory effect on zona pellucida three peptides (pZP3)-induced autoimmune POI mice, upregulating the Nrf2/HO-1/SIRT1 signaling pathway to increase Treg cell expression. In addition, icariin promoted DNA damage repair effectively attenuated ovarian damage in D-gal-induced POF mice, and significantly downregulated the expression levels of DNA damage indicators γH2AX and tumor protein p53-binding protein 1 (53BP1) (129).

#### 3.3.2 Hyperin

Quercetin-3-O-β-D-galactoside, also known as hyperin, is a flavonol glycoside primarily found in plants of the genus Chrysin and Hawthorn (130). A variety of MFH species have been used for the extraction of hyperin, including *Crataegus pinnatifida* Bunge, *Astragalus cuscutae* Bunge, *Salvia miltiorrhiza* Bunge, *Rosa rugosa* Thunb., and *Houttuynia cordata* Thunb. (131). It has been reported that a certain dose of hyperin can improve ovarian endocrine function, enhance ovarian granulosa cell viability, increase the secretion of E_2_ and P from granulosa cells, and significantly increase the expression level of CYP17 and CYP19 (132). Ma et al. (133) established a mouse model of TG-induced POI. Pathological damage to the ovaries was reduced by hyperin treatment, with increased numbers of follicles and CL, elevated serum E_2_ and AMH levels, and decreased FSH levels. The mechanism was related to the anti-oxidative stress effect of Nrf2/HO-1 pathway and the anti-apoptotic pathway of PI3K/Akt pathway. Moreover, Fang et al. (134) treated human granulosa cell line (KGN) cell with hyperin, which attenuated Triptolide-induced cellular damage and exerted anti-apoptotic effects through the Akt/tuberous sclerosis complex 1 (TSC1)/mechanistic target of rapamycin complex 1 (mTORC1) pathway.

#### 3.3.3 Puerarin

*Pueraria lobata* (Willd.) Ohwi is an essential medicinal and edible plant widely cultivated in Asian countries (135). Puerarin, also known as soyflavone-8-c-glucoside, is the main bioactive ingredient extracted from *Pueraria lobata* (136). It has a chemical structure similar to that of phytoestrogens and has been widely used clinically for its vasoprotective, antioxidant, hepatoprotective, and antiviral effects (137–140). Chen et al. (141) found that puerarin significantly increased the number of follicles and the ratio of primordial follicles, decreased the atresia ratio, upregulated the expression of mouse vasa homolog (Mvh) and octamer-binding transcription factor 4 (Oct4), and increased the levels of SOD_2_ and Nrf2 in CTX-induced POF mice. It may activate the Wnt/β-catenin signaling pathway to maintain the survival of female reproductive stem cells interfering with POF, while also alleviate oxidative stress. Other study suggested that puerarin exerted an inhibitory effect on apoptosis. The results showed that puerarin significantly reduced serum FSH and LH levels, increased E_2_ levels, and downregulated caspase-3 protein expression and regulated Bcl-2 and Bax expression in VCD-induced DOR rats (142).

#### 3.3.4 Soy isoflavones

*Sojae Semen Praeparatum* is a medicinal herb concocted from the mature seeds of the soybean (*Glycine max* (L.) Merr.) through fermentation, with soy isoflavones as the main active ingredient (143). These compounds have a similar chemical structure to endogenous estrogens, and can bind to estrogen receptors to exert both estrogenic and anti-estrogenic effects (144), helping to alleviate hormone level fluctuations and maintain ovarian reserve function (145, 146).

Soy isoflavones reduced the number of atretic follicles, lowered caspase-3 and ROS levels, and increased Bcl-2 and total antioxidant capacity (TAC) levels in naturally aged rats, which may be related to their involvement in antioxidant and anti-apoptotic processes in ovarian tissues (147). Estrogen represents a pivotal signaling molecule in this context. Its biosynthesis is regulated by the expression of the cytochrome P450 aromatase gene (CYP19A1) in ovarian and peripheral tissues, and its biological effects are primarily mediated through estrogen receptors (ERs), which play essential roles in multiple physiological processes, including reproductive homeostasis and cellular stress regulation (148). Zhang et al. (149) demonstrated that soy isoflavones could exert an estrogen-like effect in the treatment of naturally aged peri-menopausal rats by significantly increased the expression of ER-*α* in the ovary. Genistein is one of the main components of soy isoflavones (143). It has been found that genistein increased the number of primordial, secondary and sinus follicles, decreased the number of atretic follicles and delayed the cessation of the motility cycle in aged rats, thereby positively affecting the ovarian reserve function (150, 151). Zhang et al. (149) found that low doses of genistein significantly increased ER-α expression in rat ovarian granulosa cells, whereas 100 μmol/L of genistein caused a decrease in ER-α expression, confirming the dual effect of soy isoflavones on estrogen. The above studies suggested that the intervention mechanism of soy isoflavones in ovarian aging was related to antioxidant, anti-apoptotic and estrogen-like effects.

#### 3.3.5 Quercetin

Quercetin (3,3,4,5,7-pentahydroxyflavone) is a flavonoid compound widely found in MFH species, including S*emen cuscutae*, *Alpinia galanga* (L.) Willd., *Coix lacryma-jobi* L.var. *mayuen*. (Roman.) Stapf, *Polygonatum sibiricum* F. Delaroche, and *Morus alba* L. (152). This compound has various biological activities such as antioxidant, anti-inflammatory, anti-apoptosis, and improvement of mitochondrial function, thereby effectively exerting a protective effect against ovarian aging.

Chen et al. (153) showed that quercetin protected ovarian reserve function from CTX-induced damage, reducing ovarian pathology and increasing serum AMH, E_2_ and P levels, while decreasing FSH and LH levels after treatment. Mechanistically, quercetin increased ATP and mtDNA production, decreased the levels of inflammatory vesicle components (NLRP3, caspase-1, IL-1β) and gasdermin D (GSDMD), activated the peroxisome proliferator-activated receptor gamma coactivator 1-alpha (PGC1-α) pathway to reverse mitochondrial dysfunction and inhibited cellular pyroptosis. Li et al. (154) suggested that quercetin’s ability to resist apoptosis was through activation of the Janus kinase 2 (JAK2)/signal transducer and activator of transcription 3 (STAT3) signaling pathway. Another study showed that quercetin increased the antioxidant capacity and upregulated the expression of oxidative stress-related genes SOD-1, catalase (CAT), Glutathione synthetase (GSS) in the ovaries of menopausal rats (155). Guo et al. (156) found that quercetin inhibited the stromal cell-derived factor-1 (SDF-1)/C-X-C chemokine receptor 4 (CXCR4) signaling pathway to exert an anti-inflammatory effect, resulting in the improvement of ovarian pathological morphology and serum sex hormone levels in CTX-induced POI rats.

Total flavonoids from S*emen cuscutae* can prevent and intervene in ovarian aging, and quercetin is one of the main active ingredients (157). Total flavonoids from S*emen cuscutae* increased the number of follicles and CL at all levels, decreased the number of atretic follicles, elevated AMH and E_2_ levels, and decreased FSH and LH levels and the FSH/LH ratio in POF rats (157). Isoquercitrin is the glycosidic form of quercetin, which has similar biological activities and is also found in a variety of MFH species (158). Zhang et al. (159) found that treating 2,2′-azobis (2-methylpropionamidine) dihydrochloride (AAPH)-induced KGN cells with isoquercitrin increased cell viability and glutathione peroxidase (GSH-Px) enzyme activity, and reduced ROS levels. Its ability to alleviate oxidative stress was significantly better than other active ingredients in *Rubus chingii* Hu.

### 3.4 Polysaccharides

#### 3.4.1 *Dendrobium officinale* polysaccharides

*Dendrobium officinale* polysaccharides, as one of the main active components of the traditional Chinese medicine *Dendrobium nobile* Lindl., exhibit anticancer, anti-inflammatory, antioxidant, anti-aging and immunomodulatory effects (160–164). The alcoholic extract of *Dendrobium officinale* has been shown to have a good antioxidant effect, significantly increasing the number of follicular cells, increasing the activities of antioxidant enzymes such as SOD, CAT and GSH-Px and decreasing the MDA levels in D-gal-induced aging mice (165). Wu et al. (166) found that gavage treatment of naturally aged mice with *Dendrobium officinale* polysaccharides restored the ovarian index, reduced pathological ovarian damage, and increased in follicular number and E_2_ levels. *Dendrobium officinale* polysaccharide significantly reduced the levels of pro-inflammatory factors (TNF-α, IL-6) and MDA, enhanced the levels of anti-inflammatory factors (IL-10), antioxidant enzyme activities, and MMP in the ovaries, and increased the antioxidant capacity, balanced the inflammation, and improved the mitochondrial function through the inhibition of NF-κB and p53/Bcl-2 signaling pathways.

#### 3.4.2 *Lycium barbarum* polysaccharides

*Lycium barbarum* L. is the dried mature fruit of the genus Lycium in the family Solanaceae, recognized as a good source of medicine and food, with various nutrients and phytochemicals (167). *Lycium barbarum* polysaccharide is the primary active ingredient of *Lycium barbarum* L. (167). Its structure, containing the pyran ring, acidic heteropolysaccharides, and pectin contained in its structure indicate that it has good antioxidant, anti-aging, immunomodulatory, anti-tumor, and reproductive protection effects (168). Zheng et al. (169) investigated the effects of *Lycium barbarum* polysaccharide on D-gal-induced POI mice from two perspectives: intestinal flora and metabolism. The results showed that *Lycium barbarum* polysaccharide significantly increased the number of primordial follicles, primary follicles, and sinus follicles in mice, effectively regulated the disordered estrous cycle, and improved the fertility and serum FSH and LH levels. Its mechanism involved regulating pathways such as arginine biosynthesis, glycerophospholipid metabolism, and steroid hormone biosynthesis, and it was related to the intestinal flora of *Faecalibaculum, Bilophila* and *Anaerofustis* in the intestinal flora. Another study suggested that *Lycium barbarum* polysaccharide alleviated D-gal-induced POF symptoms by promoting the activation of the AMPK/SIRT1 autophagy pathway (170).

### 3.5 Terpenoids

#### 3.5.1 Catalpol

*Rehmannia glutinosa* (Gaertn.) DC. is a traditional MFH herb with the effects of nourishing yin, tonifying blood, and benefiting the essence and marrow. Catalpol is a cyclic enol ether terpene glycoside compound extracted mainly from the root of *Rehmannia glutinosa* (Gaertn.) DC., and can also be sourced from another MFH herb *Scrophularia ningpoensis* Hemsl. (171). It has been shown to possess a variety of pharmacological effects including anti-insulin, hepatoprotective, anti-inflammatory, angiogenesis-promoting, anti-tumor and anti-apoptosis (172–176). Catalpol protected the ovarian ultrastructure in naturally aged rats, improved follicle quality and quantity, significantly increased serum E_2_ and P_4_ levels, and decreased FSH and LH levels (177). Ding et al. (178) found that Catalpol effectively reduced ROS, MDA, caspase-3 and Bax levels, increased SOD, CAT and Bcl-2 levels, and inhibited oxidative stress and apoptosis by activating the Hedgehog pathway in POI rats.

#### 3.5.2 Paeoniflorin

Paeoniflorin, a monoterpene glucoside, is the primary active compound of *Paeonia lactiflora* Pall. (179), exhibiting a wide range of biological activities including neuroprotection, antidepressant effects, and immunomodulatory effects (180–183). Wu et al. (184) used cisplatin-induced DOR mice and cisplatin-induced KGN cells to investigate the effects and potential mechanisms of paeoniflorin on DOR. The treatment restored the estrous cycle, improved ovarian index and serum hormone levels, and increased numbers of sinus follicles and CL in the mice. Mechanistically, paeoniflorin promoted the expression of aromatase, a key rate-limiting enzyme for E_2_ synthesis in the ovary, activated the FSHR/cyclic adenosine monophosphate (cAMP)/protein kinase A (PKA)/cAMP response element-binding protein (CREB) signaling pathway, and restored E2 synthesis function in ovarian granulosa cells, thereby improving ovarian function.

#### 3.5.3 Tanshinone

In Chinese traditional medicine, *Salvia miltiorrhiza* Bunge is considered as a drug that activates blood circulation, removes blood stasis, calms the mind and regulates menstruation (185). Tanshinones are natural terpenoids extracted from the roots and rhizomes of *Salvia miltiorrhiza* Bunge, with main active components including tanshinone I, tanshinone IIA, dihydrotanshinone and cryptotanshinone (186). Among them, cryptotanshinone and tanshinone IIA showed significant effects in ameliorating ovarian aging. Huang et al. (187) demonstrated that cryptotanshinone improved the estrous cycle, reduced ovarian pathological damage, and normalized serum hormone levels in CTX-induced POF mice by upregulating Bax and downregulating Bcl-2, Ki-67 antigen (Ki67), and proliferating cell nuclear antigen (PCNA), thereby inhibiting granulosa cell apoptosis. Another study suggested that the effects of cryptotanshinone in alleviating oxidative stress and inhibiting apoptosis were related to the activation of the SDF-1/CXCR4 axis (188). Tanshinone IIA was found to exert antioxidant effects and upregulate the expression of antioxidant genes (CAT, Nrf2, glutathione peroxidase-1), thereby elevating AMH and E_2_ levels and promoting healthy oocyte development in naturally aged mice (189).

### 3.6 Others

#### 3.6.1 Curcumin

Curcumin, a hydrophobic polyphenol, is an important active ingredient of the Chinese herb *Curcuma longa* L. (190). Curcumin has been shown to have various biological functions including antioxidant, anti-inflammatory, anticancer, and immunomodulation effects (191–194). Lv et al. (195) found that curcumin treatment of naturally aged mice resulted in a significant increase in follicle number, a decrease in serum FSH, elevated AMH, and E_2_ levels, and a significant decrease in C-reactive protein (CRP) and IL-6 levels. It exerted anti-inflammatory effects, affected the translocation of FOXO3, and inhibited the PTEN/Akt/FOXO3a pathway, thereby protecting primordial follicles from over-activation. It has been suggested that curcumin partially exerted anti-androgenic properties, decreasing the expression of androgen receptor (AR) and cyclooxygenase-2 (COX-2) and enhancing the expression of bone morphogenetic protein 15 (BMP-15) and 3β-hydroxysteroid dehydrogenase (3β-HSD) in FSH-R haploinsufficient mice (196). Yan et al. (197) reported that curcumin inhibited oxidative stress and granulosa cell autophagy through activation of the Nrf2/HO-1 and PI3K/Akt signaling pathways, thereby improving ovarian morphology and function in D-gal-induced POF mice. Another study found that curcumin exerted antioxidant and anti-apoptotic effects to attenuate ovarian failure in naturally aged mice by increasing SIRT1 and SIRT3 gene levels (198).

#### 3.6.2 Leonurine hydrochloride

*Leonurus japonicus* Houtt. is a traditional Chinese medicine used for the treatment of gynecological disorders such as irregular menstruation, dysmenorrhea, postpartum hemorrhage and postpartum abdominal pain (199). Leonurine hydrochloride (Leo) is an alkaloid compound found only in *Leonurus japonicus* Houtt. (200), exhibiting antioxidant, anti-inflammatory, anti-apoptotic, anti-tumor, and angiogenesis-promoting activities (201–206). Leo gradually restored hormone levels in POI mice by increasing the number of primordial follicles, primary follicles, and secondary follicles, and decreasing the number of atretic follicles. Mechanistically, Leo may effectively protect mice from CTX injury by inhibiting NLRP3/GSDMD-mediated granulosa cell pyroptosis (207). Wang et al. (208) demonstrated that Leo restored the estrous cycle, regulated the disordered hypothalamic–pituitary-ovarian axis, and exerted an estrogen-like effect by elevating ER-α and ER-β expressions in mice of ovarian function decline.

## 4 Discussion and challenges

**Figure 5:**
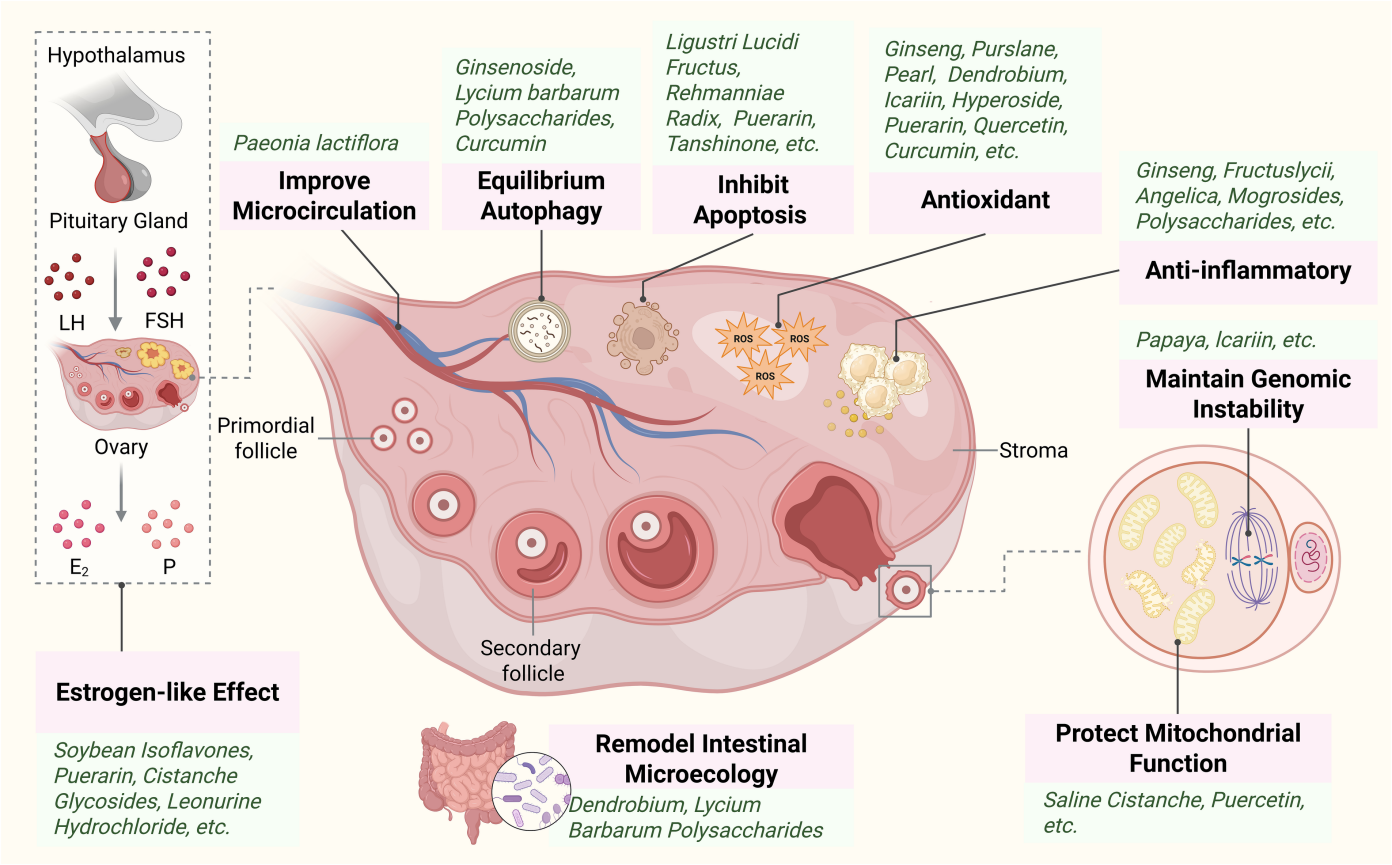
Mechanisms of MFH species and active ingredients to improve ovarian aging. MFH species extracts and ingredients (including saponins, flavonoids, polysaccharides, terpenoids, and others) exert multi-target protective effects against ovarian aging. These coordinated mechanisms collectively enhance follicular reserve, improve oocyte quality, and maintain ovarian function. LH, luteinizing hormone; FSH, follicle-stimulating hormone; E_2_, estradiol; P, progesterone; ROS, reactive oxygen species. Created in BioRender. Chen, J. (2026) https://BioRender.com/gnbt7e7.

The essence of ovarian aging is the progressive disruption of homeostasis in the ovarian microenvironment. This process commences with accelerated depletion of follicular reserves and diminished oocyte function, ultimately triggering reproductive endocrine disorders through systemic interactions and elevating long-term health risks in women. The concept of medicinal foods has existed within traditional Chinese medicine for centuries, yet only recently garnered attention from scientific communities and the food industry. This review synthesizes existing research evidence, indicating that medicinal plants primarily delay ovarian aging through three pathways: (1) mitigating oxidative stress via the Nrf2/ARE, Sirt1, AKT/FOXO3, and Wnt/β-catenin pathways; (2) reducing inflammatory responses through the NF-κB and NLRP3 pathways; (3) inhibition of apoptosis and autophagy via JAK2/STAT3, SDF-1/CXCR4, and PI3K/Akt pathways. Additionally, they exert effects by promoting DNA damage repair, modulating the hypothalamic–pituitary-ovarian axis, oestrogen receptors, and gut microbiota to regulate reproductive hormone levels. In summary, there are more types and studies on flavonoid active ingredients, while fewer studies have focused on alkaloid compounds. The various compounds showed significant differences in their mechanisms of action due to structural differences (Figure 5). Saponins focus on anti-inflammatory effects, flavonoids components prefer antioxidant effects, terpenoids and polyphenols focus on antioxidant and anti-apoptotic effects, and polysaccharides are characterized by intestinal flora regulation. Moreover, puerarin, quercetin, and soy isoflavones—which are phytoestrogens with estrogen-like effects—also play significant roles in mitigating ovarian aging. Based on these characteristics, different ingredients can be rationally combined to promote overall ovarian health.

Overall, the included studies exhibit substantial methodological heterogeneity—including differences in animal species, induction models (D-gal, VCD, CTX, natural aging), extract preparations, dosages, treatment duration, and outcome indicators—which limits direct comparability. Chemically induced models reproduce specific aspects of ovarian injury but lack full relevance to human ovarian aging, whereas natural aging models increase physiological validity yet introduce greater variability. Despite these differences, most studies consistently report improvements in hormone profiles, follicle numbers, and oxidative-stress markers, although the magnitude of therapeutic benefit varies considerably across models and experimental designs. Meanwhile, few studies reported standardized characterization of herbal extracts, hindering reproducibility and reliable comparison across experiments.

As of 2019, the size of the national health food market, including the output value of MFH products, had exceeded 300 billion yuan, with an annual growth rate of 14%. Classical Chinese medicine compound formulas such as Zuogui Pills (209), Yangjing Zhongyu Decoction (210), and Si-Wu-tang (211), which are widely used with MFH species, have systematic advantages in improving ovarian function, and are expected to be an important source of modern functional preparations in the future. However, the application of MFH herbs in interventions targeting ovarian aging still faces several significant challenges. First, although these plants are widely consumed as dietary components, most current evidence is limited to animal studies and *in vitro* models; robust clinical trials evaluating single MFH herbs or their bioactive constituents remain scarce. Second, despite the relatively abundant clinical studies on classical multi-herb formulas in traditional Chinese medicine, these prescriptions are often highly complex and frequently extend beyond the MFH category. How to fully harness the synergistic and potentiating effects of pure MFH-based formulations requires further systematic investigation. Third, the mechanisms of action for most MFH herbs and their active compounds remain insufficiently defined. Key molecular targets and signaling pathways have yet to be elucidated, and it is still unclear whether the gut-ovary axis plays a critical regulatory role in their therapeutic effects. Fourth, although MFH plants are considered natural and generally safe, long-term use may still pose risks, including dose-dependent toxicity or potential endocrine-modulating effects. Therefore, their safe dosage ranges, treatment duration, possible adverse effects, and suitable populations must be clearly established. Finally, the heterogeneous sources and variable quality of herbal materials highlight the urgent need to develop rigorous, standardized quality-control systems and harmonized manufacturing practices for MFH products. Most existing studies, including those discussed in this review, focus on ovarian phenotypic and functional outcomes rather than tissue-specific distribution or pharmacokinetics. Future studies incorporating tissue distribution and ovary-specific delivery strategies are needed to further clarify this issue.

## 5 Conclusion

This review comprehensively summarized the MFH species and active ingredients (saponins, flavonoids, polysaccharides, terpenoids, and others) with ameliorative properties in ovarian aging in the published literature, and summarizes the sources, experimental models, efficacy and potential mechanisms. The MFH species and active ingredients primarily intervened in ovarian aging by reducing oxidative stress, inhibiting apoptosis, balancing autophagy, anti-inflammation, regulating mitochondrial function, and estrogen-like effects, and were characterized by multiple pathways and targets. In the future, with in-depth research on the mechanism, advancement of clinical trials and the emergence of technological innovations, it is expected that more new types of medicines or healthcare products will be developed with low-toxicity, widely sourced and renewable MFH species and active ingredients as research subjects, thereby providing richer choices for women’s health.
